# Mindfulness‐Based Cognitive Defusion Training for Perceived Stress in Older Adults with Mild Cognitive Impairment: A Randomized Controlled Study

**DOI:** 10.1002/alz.088248

**Published:** 2025-01-09

**Authors:** Yinxia Ren, Lulu Shi, Chenxi Ge, An Gu, Lina Wang

**Affiliations:** ^1^ Huzhou University, Huzhou, Zhejiang China

## Abstract

**Background:**

Recognizing perceived stress as a modifiable cognitive risk factor, mindfulness‐based programs emerge as promising for stress mitigation in older adults with Mild cognitive impairment (MCI). However, existing research, primarily observational and focused on chronic patients and caregivers, necessitates developing and evaluating MCI‐specific mindfulness interventions.

**Design:**

A two‐arm and assessor‐blinded randomized controlled trial.

**Settings and participants:**

Community‐dwelling older adults with MCI.

**Method:**

Participants (N = 102) were randomly allocated to one of the two arms: (1) a M‐bCDT program (weekly 60‐minute supervised sessions for 8 weeks, followed by 12 weeks of unsupervised practice) or (2) health promotion classes alone, with M‐bCDT program after the follow‐up assessment. Measures of perceived stress levels, cognitive function, and mindfulness awareness levels were collected at baseline (T0), 8‐week (T1), and 20‐week (T2). Intervention effects were assessed both at a group level (Generalized Estimating Equation, GEE) and at an individual level (Reliable Change Index, RCI).

**Result:**

In the intervention group, the M‐bCDT program demonstrated significant interaction effects compared to the waitlist control group. GEE analysis revealed showed statistically significant effects on perceived stress at both measured intervals (β_T1_ = ‐3.686, P<0.001; β_T2_ = ‐7.608, P<0.001). In cognitive functioning, significant cognitive improvements were noted in the Finger Tapping test (β_T1_ = 1.020, P = 0.026; β_T2_ = 3.000, P<0.001), Purdue Pegboard Test Assembly and Bimanual Tasks (β_T1_ = 0.549/0.784, P = 0.048/0.013; β_T2_ = 1.510/2.059, P<0.001), Auditory Verbal Learning Test (AVLT‐immediate/delayed recall: β_T1_ = 1.098/0.667, P<0.001/0.038; β_T2_ = 2.176/1.373, P<0.001), and Montreal Cognitive Assessment scores, mindfulness awareness levels in observation and non‐judgment dimension improved significantly over time. RCIs indicated improvements in the intervention group of 1.96% in perceived stress level, 70.59% and 60.78% in AVLT‐immediate/delayed recall after 8 weeks, which further increased to 31.37%, 90.20%, and 78.43% after 20 weeks.

**Conclusion:**

The M‐bCDT program effectively reduced the perceived stress levels in older adults with MCI, facilitating the improvements in cognitive function and mindfulness awareness levels, with sustained benefits for three months. It offers a standardized approach for community healthcare providers in MCI cognitive management.

**Trial registration:**

The trial was registered at Chinese Clinical Trials Registry on 05/18/2020 (www.chictr.org.cn; Number Registry: ChiCTR2000033013).

**Funding:**

National Natural Science Foundation of China (NO. 72174061; NO. 71704053), China Scholarship Council Foundation (NO. 202308330251).

